# State Registration: A Jeremiad

**Published:** 1919-04-12

**Authors:** 


*To the Editor of* The Hospital.

### STATE REGISTRATION: A JEREMIAD.

*To the Editor of* The Hospital.

*She asked bread, and they gave her a stone.*

Sir,—May I claim the hospitality of your Letter-Box, where all and sundry are permitted to congregate and to give vent to their feelings, whether they belong to the orthodox schools or stand shivering outside their respectable doors? The joy-bells are ringing everywhere; the feast is spread, for it is Der Tag. The dream of thirty years is being realised, and the British Parliament is enacting a measure for the State Registration of Nurses. And while the jubilation proceeds I am asking permission to have a little corner all to myself where I can make my lamentation. I do not know whether this is a sign of lunacy or not; it would certainly seem like it, for what appears to everybody obvious must be right and true.

Nevertheless, I am in mourning. I have a feeling, amounting to a conviction, that the nurses' birthright is being sold for a mess of pottage, and that they will one day awake to discover that what now looks so fair is nothing but Dead Sea fruit. If I should by any remote possibility happen to be right, I am sure the reader will agree that it is a proper occasion for mourning and lamentation. Since the days of Florence Nightingale there never has been such a golden opportunity for the emancipating of the British nurse as this. The nation was never at any time so willing to respond to any reasonable demand made on her behalf, a fact made plain by Parliament and the Press. Until now the nurse's claims have never been presented to the public, a fact which is scarcely realised even in well-informed circles. It was in no vague manner that the nation responded to the call of the nurse after the Crimea. Fifty thousand pounds was the thank-offering of a grateful country—a sum equivalent in purchasing power to at least a million to-day.

Please observe, I am in no respect opposed to State Registration. On the contrary, I can appreciate that it has its advantages. If all the trained nurses in the country register there will be an available list, with names and addresses. My lamentation, therefore, is not because the Bill is successfully passing through Parliament, but for quite another reason. Parliament and the nation are persuaded that herein is the salvation and emancipation of the nurse, her reward for faithful service, her joy and crown. This conclusion is and has been inevitable. For thirty years it has been dinned into the ears of everybody that State Registration is the elixir of life, and during those thirty years every real reform has been neglected. It would be a miracle if this false doctrine preached by nurses themselves should

### THE EDITOR'S LETTER-BOX—(*continued*).

not be believed by members of Parliament and echoed in the lay press. It is not so long since a well-known Dublin physician, Dr. Percy Kirkpatrick, wrote a pamphlet in which he stated that when this Bill, so often presented, became law, it would become penal for any other than a registered nurse to tend the sick for gain. If this were so, then there would be solid justification for rejoicing. But it is not so, and no Bill ever presented to Parliament on behalf of nurses contained such a provision. **I affirm, and I challenge denial, that this enactment cannot by any possibility improve the prospects of the trained nurse. It cannot have the effect of widening her field of operations, increasing her emoluments, shortening her hours of toil, providing for her age or infirmity.** It is some years since the teaching profession obtained a similar Act of Parliament. No doubt it had some small utility, but it did not prevent the teachers' strike in Wales! It does not prevent the wholly ignorant and unlettered from setting up private or public seminaries "for the sons of gentlemen" wheresoever they choose. Nurses' State Registration may be suitably compared with a signet ring on the finger of a man or woman. It is sometimes useful, somewhat expensive, but it does not increase efficiency, or comfort, or affect social status. If a man or woman had rendered invaluable services to the nation, and saved numberless lives during the war, the award of a signet ring would fall little short of being an insult.

I consider that the women who have ridden this State Registration horse to the death have done the cause of nurses great if not altogether irreparable damage. The nurse wanted bread. They have demanded for her and received a stone. They have sold her birthright. I do not know whether it is too late to remedy the evil or not. I do not know whether any champion will arise and tell the British public the truth. Even if the attempt is made it will be difficult to accomplish effectually. Everywhere in the newspapers I read how at last the nurse is receiving her guerdon, how, after years of agitation, this measure of bare justice has been wrung from the law-givers. It will take a great deal of propaganda to dispel the illusion thus created, and hence my lament. I note that it is proposed in Parliament that nurses themselves are to pay for the administration of the Bill. It is suggested that if 50,000 register at two guineas apiece there will be enough to defray the cost of maintaining a Council, a staff, and keeping the roll of membership up to date. I should think that an investment in War Bonds would be more profitable for those who have more guineas than they know what to do with.

The only drop of consolation that I can derive from this nursing Der Tag is that it will come to an end. I remember some years ago reading a comment in *Truth* as to a certain date. *Truth* looked forward with delight to the morrow of that date. A certain encyclopædia was being boomed and advertised in the press, by circular, and by telegram. Up to a certain date it was on the market at a low price, and then the offer was to be withdrawn. When the day is over, commented *Truth*, we shall have a little peace and quietness in our lives once more. And so I am glad that these State Registrationists are being given their toy. We shall have one terror to existence removed when their agitation comes to an end. But what of the nurse? How long will she take to realise that all the rejoicing is only because another council has been set up, and that her own hands are still empty?—Yours, &c.,

Ierne.

